# External validation and comparative performance of four risk scores for acute kidney injury in critically ill adults

**DOI:** 10.1080/0886022X.2026.2728524

**Published:** 2026-10-15

**Authors:** Fangyan Liu, Linpei Jia, Jing Wang, Lanjing Wang, Zheng Wang, Dandan Zhu, Meng Wang, Zhi Liu, Sijie Li

**Affiliations:** a Department of Emergency, Xuanwu Hospital, Capital Medical University, Beijing, China; b National Clinical Research Center for Geriatric Disorders, Advanced Innovation Center for Human Brain Protection, Capital Medical University, Beijing, China; c Trade union office, Xuanwu Hospital, Capital Medical University, Beijing, China; d Department of Nephrology, Xuanwu Hospital, Capital Medical University, Beijing, China; e Neuro Cardio Vascular Diseases Center, Xuanwu Hospital, Capital Medical University, Beijing, China; f Department of Neurology, Xuanwu Hospital, Capital Medical University, Beijing, China

**Keywords:** Emergency, intensive care, risk assessment, acute kidney injury, prognosis

## Abstract

**Background:**

Several parsimonious scores predict acute kidney injury (AKI), but their comparative performance in critically ill adults is uncertain. We hypothesized that the ICU-derived UCSD-Mayo score would show higher discrimination than three scores developed in broader acute-care populations.

**Methods:**

This single-center retrospective validation study included 1,723 emergency ICU patients admitted from 2014 to 2021. We compared the UCSD-Mayo, AKI-RiSc, Bell, and APS scores for in-hospital AKI, AKI within 7 days, and stages 2 and 3 AKI. Analyses included AUROC with paired DeLong comparisons, cross-validated local recalibration, calibration plots, IDI, categorical NRI at the event rate, decision-curve analysis, and prespecified sensitivity analyses.

**Results:**

In-hospital AKI occurred in 406 patients (23.6%), 281 (16.3%) developed AKI within 7 days, and 169 (9.8%) developed stages 2 and 3 AKI. UCSD-Mayo had the highest observed AUROC for in-hospital AKI (0.713, 95%CI 0.685–0.740), 7-day AKI (0.690, 0.658–0.723), and stages 2 and 3 AKI (0.706, 0.665–0.746). Its 7-day AUROC did not differ significantly from AKI-RiSc (difference 0.029, 95%CI −0.006 to 0.064; *p* = 0.103). Reclassification and net benefit varied by outcome and threshold; sensitivity analyses did not materially change the overall pattern.

**Conclusions:**

In this single-center Chinese emergency ICU cohort, UCSD-Mayo showed the highest observed discrimination, but improvements were modest and were not uniform across all outcomes, metrics, or decision thresholds. These scores may support risk awareness and monitoring but should not be used as stand-alone treatment rules.

## HIGHLIGHTS

1. UCSD-Mayo showed the highest observed AUROC, with modest and outcome-dependent differences from comparator scores.
2. Calibration, reclassification, and decision-curve analyses showed that comparative performance depended on outcome and risk threshold.

## Introduction

Acute kidney injury (AKI) is frequent in critical illness and is associated with mortality and prolonged hospitalization [1–4]. Survivors, particularly after dialysis-requiring AKI, remain at increased risk of chronic kidney disease, chronic dialysis, and death [5–7]. Because serum creatinine rises after injury has occurred, prediction tools may help clinicians identify patients who warrant closer kidney-function surveillance before AKI is clinically apparent.

Electronic alerts and clinical decision support have been evaluated to improve AKI recognition [8,9], while unequal access to kidney replacement therapy reinforces the importance of prevention and early management [10]. Numerous injury biomarkers have also been studied [11–19], but routine availability and transportability remain limited. Prediction-model performance likewise changes across settings and populations, making direct comparison in the intended target population necessary before implementation.

We selected four point-based scores because every component could be reconstructed from routinely collected admission data in our electronic records, the scores represented distinct target settings, and each had a published, interpretable scoring rule: UCSD-Mayo for ICU patients [20], AKI-RiSc for emergency admissions [21], Bell for the general population [22], and APS for acute medical admissions [23]. This was a pragmatic, prespecified comparison rather than a systematic review of all published AKI models; more complex ICU tools such as AKIpredictor were not reconstructable from the archived variables.

Our objective was to externally validate and compare the four scores in Chinese emergency ICU patients. We hypothesized that UCSD-Mayo, the only score developed specifically in an ICU population, would have higher discrimination; we also evaluated calibration after local score-to-risk mapping, reclassification, and net clinical benefit to determine whether any observed advantage was clinically meaningful.

## Methods

### Study design and participants

This single-center retrospective cohort study was conducted in the Emergency Intensive Care Unit (EICU) of Xuanwu Hospital, Capital Medical University. Consecutive admissions from 1 January 2014 to 31 December 2021, were screened. No patient had COVID-19. The study is reported in accordance with the TRIPOD 2015 prediction-model validation statement [24], and the official validation-only checklist accompanies the revision.

Patients were excluded for fewer than two serum creatinine (sCr) measurements during hospitalization, advanced kidney dysfunction at admission (sCr ≥353.6 μmol/L), age <18 years, or missing core data needed to calculate any score. Of 2,743 screened patients, 1,020 (37.2%) met at least one exclusion criterion and 1,723 were analyzed. Contemporaneous screening records reported 396 with fewer than two sCr measurements, 217 with advanced kidney dysfunction, 31 aged <18 years, and 393 with missing core score data; exclusion categories overlapped and therefore are not additive. Individual-level records for excluded patients are no longer retrievable, precluding valid multiple imputation of the excluded cohort.

The Ethics Committee of Xuanwu Hospital approved this study (approval No. [2025]271-001) and waived the requirement for individual informed consent. The waiver was granted because this was a retrospective, observational analysis of existing electronic medical records that involved no prospective intervention, no patient contact, and no collection of new biological specimens. The study posed no more than minimal risk: all data were de-identified and anonymized before analysis, and no identifiable patient information is reported in this manuscript. Therefore, the waiver does not adversely affect the rights or welfare of the participants. The study was conducted in accordance with the Declaration of Helsinki.

### Data collection

Demographic, clinical, and laboratory variables were extracted from the electronic record. Unless specified otherwise, the first measurement within 24 h of EICU admission was used. The kidney-function component was based on admission eGFR calculated from the first admission creatinine using CKD-EPI; it reflects reduced admission eGFR rather than a confirmed chronic CKD diagnosis. Comorbidities were identified by ICD-10 codes. AVPU was classified as Alert, responsive to Verbal stimulus, responsive to Pain, or Unresponsive; APS assigned points for any category other than Alert. Anemia was defined as hemoglobin <9 g/dL (90 g/L) or hematocrit <27%.

Nephrotoxic agents comprised amphotericin B, aminoglycosides, radiocontrast, chemotherapy, antiretroviral drugs, and nonsteroidal anti-inflammatory drugs other than aspirin. Exposure was defined as radiocontrast use, concurrent use of at least three nephrotoxins, or aminoglycoside therapy for at least 3 days.

Four total scores were calculated according to their published point systems (Supplemental Tables 1-4). Predictor timing and definitions differed from some development studies: UCSD-Mayo evaluated acute factors within 48 h, AKI-RiSc and APS used acute-admission variables for 7-day outcomes, and Bell predicted 1-year risk in the general population. These differences were considered when interpreting transportability.

**Table 1.** Characteristics of the study cohort.

Variables	Whole cohort (*n* = 1,723)	AKI (*n* = 406)	Non-AKI (*n* = 1,317)
Male, No. (%)	937 (54%)	216 (53%)	721 (55%)
Age, median (IQR), y	77 (65, 83)	78 (65, 84)	77 (65, 83)
Height, median (IQR), cm	165 (160, 171)	165 (160, 172)	165 (160, 171)
Weight, median (IQR), kg	65 (55, 75)	65 (54, 75)	65 (55, 75)
BMI, median (IQR), kg/m²	24.22 (20.98, 27.34)	23.31 (20.70, 26.67)	24.36 (21.47, 27.35)
Respiratory rate, median (IQR), breaths/min	22 (19, 25)	21 (19, 26)	22 (19, 25)
Pulse rate, median (IQR), beats/min	90 (78, 104)	93 (80, 107)	89 (77, 102)
Diastolic blood pressure, median (IQR), mm Hg	69 (60, 80)	67 (58, 78)	70 (61, 80)
Systolic blood pressure, median (IQR), mm Hg	130 (114, 146)	127 (109, 145)	130 (116, 146)
AVPU (alert)	1,506 (87%)	328 (81%)	1,178 (89%)
First sCr after admission, median (IQR), μmol/L	85 (63, 125)	103 (70, 164)	81 (61, 115)
Admission eGFR, median (IQR), mL/min/1.73 m²	69 (43, 92)	51 (28, 84)	72 (48, 94)
Hb, median (IQR), g/L	113.0 (95.0, 128.0)	105.0 (88.0, 122.0)	115.0 (100.0, 130.0)
pH, median (IQR)	7.42 (7.37, 7.45)	7.40 (7.33, 7.45)	7.42 (7.38, 7.45)
Bicarbonate, median (IQR), mmol/L	24.50 (22.10, 27.50)	23.40 (20.30, 26.60)	24.80 (22.60, 27.60)
Admission eGFR <60 mL/min/1.73 m², *n* (%)	727 (42%)	242 (60%)	485 (37%)
Hypertension, *n* (%)	1,143 (66%)	258 (64%)	885 (67%)
Diabetes, *n* (%)	747 (43%)	175 (43%)	572 (43%)
Congestive heart failure, *n* (%)	736 (43%)	216 (53%)	520 (40%)
Chronic liver disease, *n* (%)	180 (10%)	54 (13%)	126 (10%)
Atherosclerotic coronary disease, *n* (%)	1,150 (67%)	291 (72%)	859 (65%)
Severe infection/sepsis, *n* (%)	764 (44%)	219 (54%)	545 (41%)
Mechanical ventilation, *n* (%)	572 (33%)	239 (59%)	333 (25%)
Nephrotoxin exposure during hospitalization, *n* (%)	169 (10%)	42 (10%)	127 (10%)
UCSD-Mayo risk score, median (IQR), score	7 (4, 9)	9 (7, 11)	6 (4, 8)
AKI-RiSc, median (IQR), score	3 (2, 4)	4 (2, 7)	2 (2, 4)
Bell risk score, median (IQR), score	13 (9, 18)	16 (11, 24)	12 (9, 17)
APS, median (IQR), score	5 (4, 7)	6 (4, 7)	5 (4, 6)
AKI within 7 days	281 (16%)	281 (69%)	0 (0%)
AKI stage			
0	1,317 (76%)	0 (0%)	1,317 (100%)
1	237 (14%)	237 (58%)	0 (0%)
2	60 (4%)	60 (15%)	0 (0%)
3	109 (6%)	109 (27%)	0 (0%)
Hospital mortality, *n* (%)	272 (16%)	185 (46%)	87 (7%)
Hospital stay, median (IQR), days	11 (8, 15)	12 (7, 18)	11 (8, 15)
ICU stay, median (IQR), days	8 (5, 13)	11 (6, 17)	8 (5, 12)

Abbreviations: IQR, interquartile range; BMI, body mass index; AVPU, Alert, Verbal, Pain, Unresponsive; sCr, serum creatinine; eGFR, estimated glomerular filtration rate; Hb, hemoglobin.

**Table 2.** Discrimination of the four scores for in-hospital AKI.

Score	AUROC	95%CI	AUROC difference vs UCSD-Mayo (95% CI)	*p*
UCSD-Mayo	0.713	0.685–0.740	Reference	*-*
AKI-RiSc	0.634	0.602–0.665	0.079 (0.047 to 0.111)	<0.001
Bell	0.635	0.603–0.667	0.077 (0.048 to 0.107)	<0.001
APS	0.598	0.568–0.628	0.115 (0.085 to 0.145)	<0.001

**Table 3.** Discrimination of the four scores for AKI within 7 days.

Score	AUROC	95%CI	AUROC difference vs UCSD-Mayo (95%CI)	*p*
UCSD-Mayo	0.690	0.658–0.723	Reference	*-*
AKI-RiSc	0.661	0.626–0.696	0.029 (−0.006 to 0.064)	0.103
Bell	0.648	0.612–0.684	0.042 (0.009 to 0.075)	0.012
APS	0.599	0.564–0.635	0.091 (0.056 to 0.125)	<0.001

**Table 4.** Discrimination of the four scores for stages 2 and 3 AKI.

Score	AUROC	95%CI	AUROC difference vs UCSD-Mayo (95%CI)	*p*
UCSD-Mayo	0.706	0.665–0.746	Reference	*-*
AKI-RiSc	0.646	0.603–0.690	0.059 (0.016 to 0.103)	0.008
Bell	0.634	0.587–0.681	0.072 (0.031 to 0.113)	<0.001
APS	0.562	0.517–0.606	0.144 (0.104 to 0.184)	<0.001

### Outcome measures

The primary outcome was in-hospital AKI of any stage. Secondary outcomes were AKI within 7 days and stages 2 and 3 AKI during hospitalization. AKI was determined dynamically from all available sCr measurements: an absolute increase ≥0.3 mg/dL (≥26.5 micromol/L) within 48 h or an increase to ≥1.5 times the value 7 days earlier. The first time either criterion was met defined AKI onset; staging followed KDIGO sCr criteria [25]. Urine-output criteria were unavailable.

### Statistical analysis

Categorical variables are reported as *n* (%) and continuous variables as mean (standard deviation) or median (interquartile range), as appropriate. Discrimination was quantified by AUROC with 95% confidence intervals; correlated AUROCs were compared using the paired DeLong method [26]. Threshold-dependent measures at the Youden cut point [27] were prespecified as descriptive analyses and are reported in the Results section and Supplementary Material; they should not be interpreted as treatment thresholds.

Because the four point scores use different prediction horizons and do not all provide directly comparable absolute-risk equations, cross-validated local calibration metrics were obtained from stratified 10-fold out-of-fold logistic mappings of each score to outcome probability [28,29]. We report calibration slope, calibration-in-the-large, observed-to-expected ratio, and Brier score from these mappings. For the visual calibration plots, we retained the original apparent-calibration analysis: outcome-specific logistic models fitted in the full cohort, with each score as the sole predictor, provided the predicted probabilities for decile observed rates, LOESS smooths, and probability histograms. These analyses distinguish cross-validated local score-to-risk metrics from apparent calibration plots and are not strict external calibration of the original absolute-risk equations. Incremental performance of UCSD-Mayo was summarized by integrated discrimination improvement (IDI) and categorical net reclassification improvement (NRI) at each outcome’s observed event rate. Decision-curve analysis compared all four scores with treat-all and treat-none strategies across clinically plausible thresholds.

Analyses used the 1,723-patient score-complete cohort; no score or outcome value was missing. Height (*n* = 8), weight (*n* = 7), BMI (*n* = 10), and blood pressure (*n* = 2) had missing descriptive values and were summarized using available observations. Sensitivity analyses (1) set the whole-stay nephrotoxin component to zero, (2) restricted 7-day AKI analysis to patients surviving at least 7 days, and (3) stratified 2014 to 2019 versus 2020 to 2021. Calibration-metric confidence intervals used 1,500 bootstrap resamples; in the calibration plots, binned observed-rate confidence intervals were Wilson 95% confidence intervals and LOESS bands were 95% confidence limits. Reclassification and sensitivity-analysis confidence intervals used 5,000 resamples. Analyses were conducted using both Python 3.13 and SAS version 9.4.

## Results

### Characteristics of study participants

Among 2,743 screened admissions, 1,020 (37.2%) met at least one exclusion criterion and 1,723 formed the score-complete analysis cohort (Supplemental Figure 1). Core score components and all three AKI outcomes were complete in the analysis cohort. Missing descriptive values were limited to height (8), weight (7), BMI (10), and blood pressure (2). Median age was 77 years (IQR 65–83), 937 (54.4%) were men, and 272 (15.8%) died in hospital (Table 1). In-hospital AKI occurred in 406 (23.6%): stage 1 in 237 (13.8%), stage 2 in 60 (3.5%), and stage 3 in 109 (6.3%). AKI occurred within 7 days in 281 (16.3%), and stage 2 and 3 AKI occurred in 169 (9.8%). Admissions were predominantly for cardiovascular (*n* = 785, 45.6%) or respiratory (*n* = 611, 35.5%) diseases, with other categories detailed in Supplemental Table 5.

### Performance of risk prediction scores

#### Performance of the UCSD-Mayo risk score, AKI-RiSc, Bell risk score, and APS in predicting in-hospital AKI

For in-hospital AKI, AUROCs were 0.713 (95%CI 0.685–0.740) for UCSD-Mayo, 0.634 (0.602–0.665) for AKI-RiSc, 0.635 (0.603–0.667) for Bell, and 0.598 (0.568–0.628) for APS (Figure 1; Table 2). Compared with UCSD-Mayo, AUROC differences were 0.079 for AKI-RiSc, 0.077 for Bell, and 0.115 for APS (all *p* < 0.001) (Supplemental Table 6).

**Figure 1.**
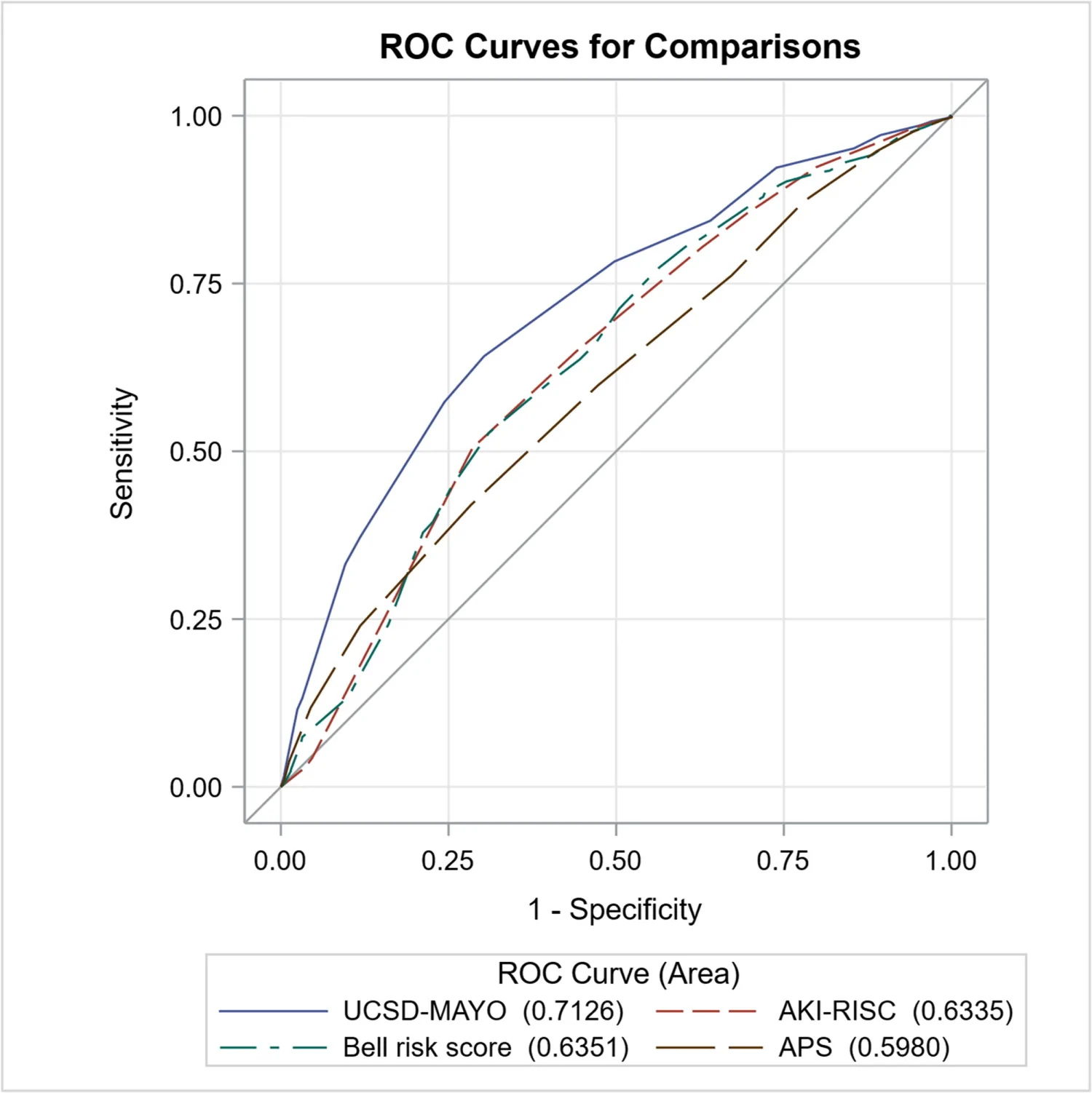
ROC curves comparing the ability of the UCSD-Mayo risk score, AKI-RiSc, Bell risk score, and APS to predict in-hospital AKI.

#### Performance of the UCSD-Mayo risk score, AKI-RiSc, Bell risk score, and APS in predicting AKI within 7 days of admission

For AKI within 7 days, AUROCs were 0.690 (95%CI 0.658–0.723) for UCSD-Mayo, 0.661 (0.626–0.696) for AKI-RiSc, 0.648 (0.612–0.684) for Bell, and 0.599 (0.564–0.635) for APS (Figure 2; Table 3). The difference between UCSD-Mayo and AKI-RiSc was 0.029 (95%CI −0.006 to 0.064; *p* = 0.103); differences versus Bell and APS were 0.042 (*p* = 0.012) and 0.091 (*p* < 0.001), respectively (Supplemental Table 7).

**Figure 2.**
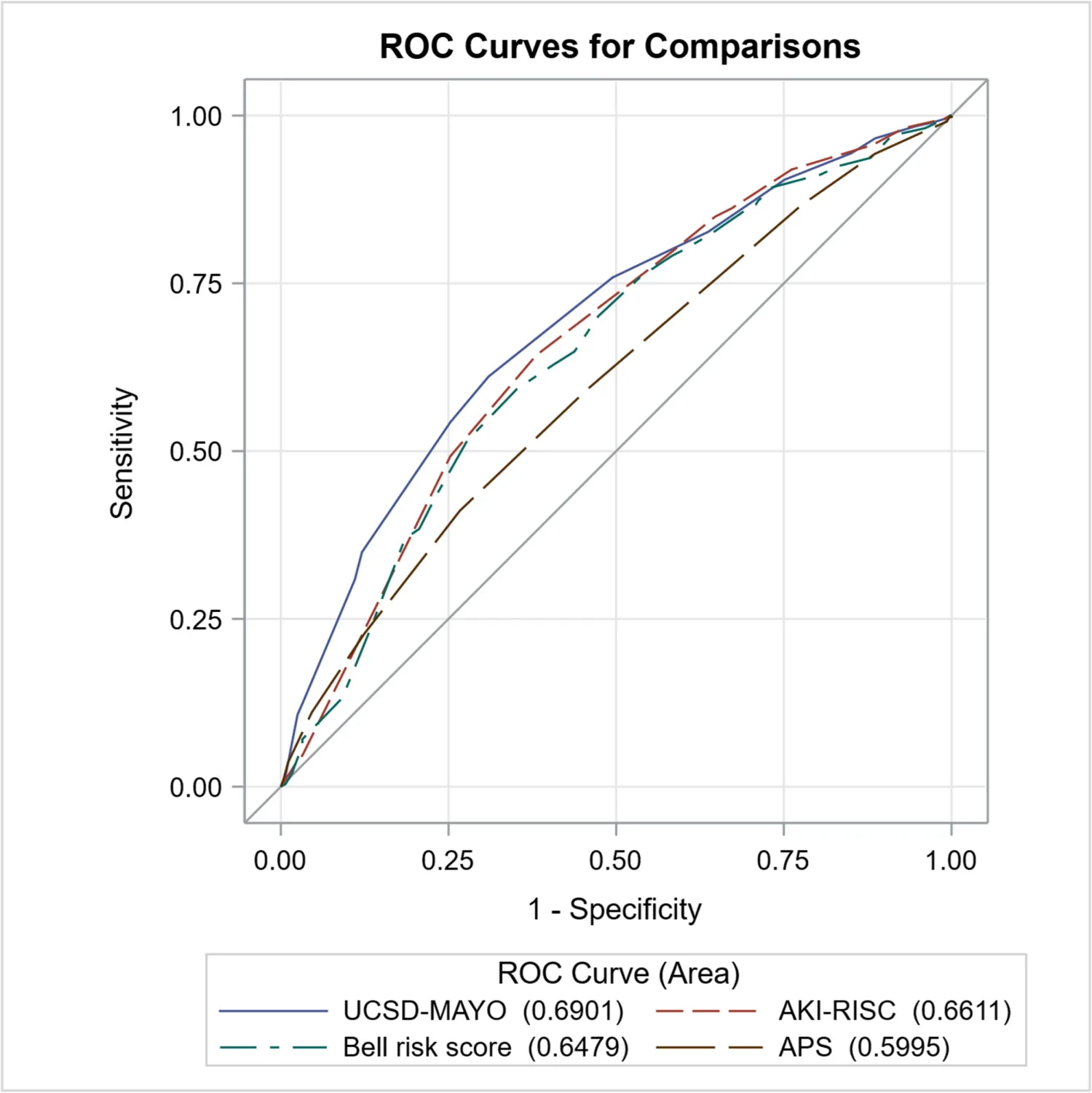
ROC curves comparing the ability of the UCSD-Mayo risk score, AKI-RiSc, Bell risk score, and APS to predict AKI within 7 days of admission.

#### Performance of the UCSD-Mayo risk score, AKI-RiSc, Bell risk score, and APS in predicting more severe AKI (stages 2 and 3)

For stages 2and 3 AKI, AUROCs were 0.706 (95%CI 0.665–0.746) for UCSD-Mayo, 0.646 (0.603–0.690) for AKI-RiSc, 0.634 (0.587–0.681) for Bell, and 0.562 (0.517–0.606) for APS (Figure 3; Table 4). AUROC differences versus UCSD-Mayo were 0.059 (*p* = 0.008), 0.072 (*p* = 0.001), and 0.144 (*p* < 0.001), respectively (Supplemental Table 8).

**Figure 3.**
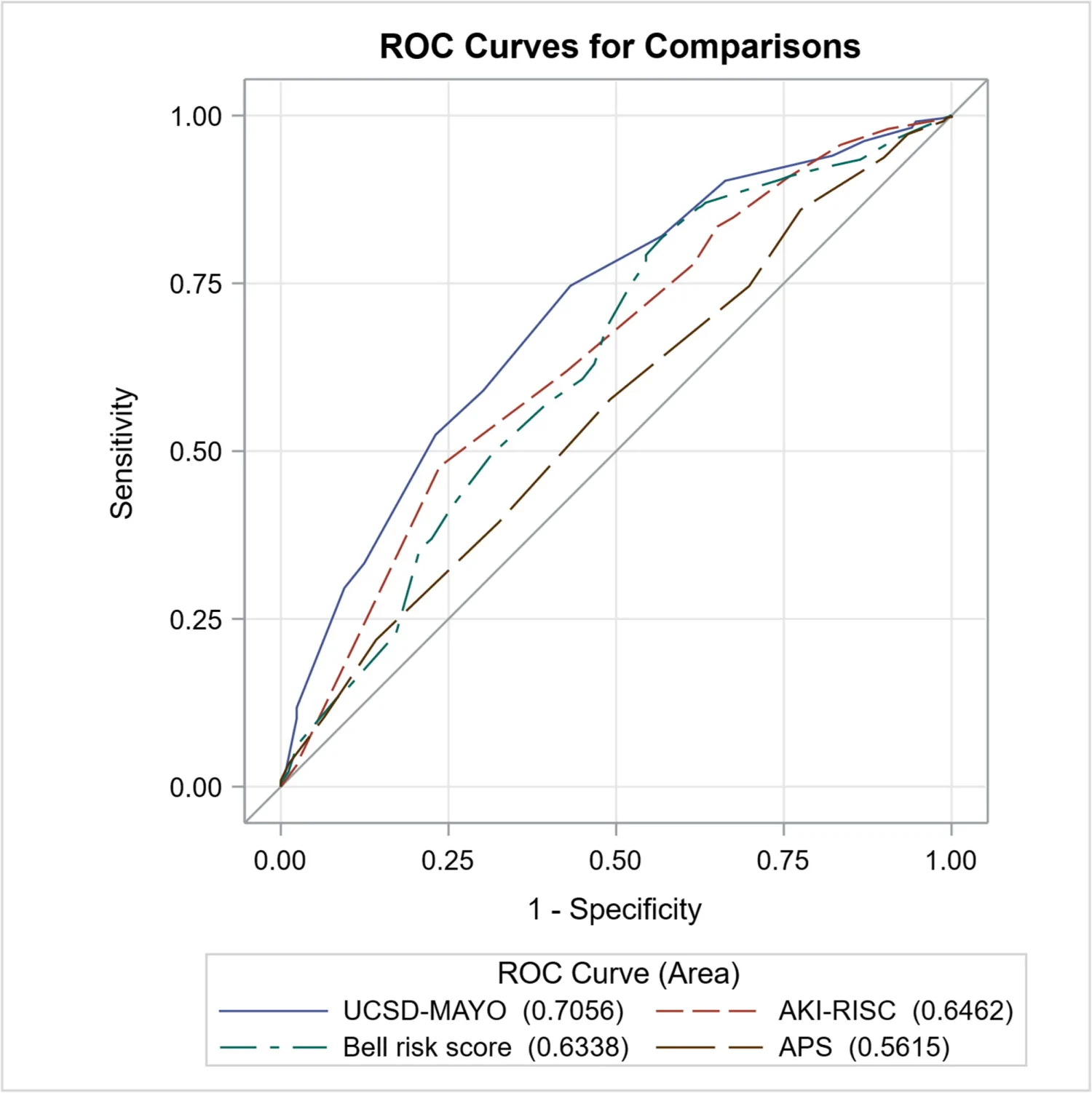
ROC curves comparing the ability of the UCSD-Mayo risk score, AKI-RiSc, Bell risk score, and APS to predict more severe AKI (Stages 2 and 3).

#### Calibration, reclassification, clinical utility, and sensitivity analyses

Cross-validated local calibration metrics showed calibration slopes ranging from 0.979 to 0.990 for in-hospital AKI, 0.968 to 0.984 for 7-day AKI, and 0.929 to 0.994 for stages 2 and 3 AKI; calibration-in-the-large values were close to zero and O/E ratios close to 1.00 (Supplemental Table 9). Separate apparent calibration plots based on full-cohort score-to-risk mappings are shown in Supplemental Figures 2-4. At event-rate thresholds, categorical NRI for UCSD-Mayo versus the three comparators ranged from 0.130 to 0.213 for in-hospital AKI, 0.087 to 0.166 for 7-day AKI, and 0.113 to 0.202 for stages 2 and 3 AKI. IDI was positive except for the 7-day comparison with AKI-RiSc, whose 95%CI included zero (Supplemental Table 10). Decision curves showed threshold-dependent net benefit: UCSD-Mayo generally had the highest net benefit at 10% to 40% for in-hospital AKI, at 10% to 20% for 7-day AKI, and at 5% to 10% for stages 2 and 3 AKI; other scores were similar or slightly higher at selected thresholds (Supplemental Figures 5-7).

Nephrotoxin exposure was recorded in 169 patients (9.8%). Setting this whole-stay component to zero changed UCSD-Mayo AUROC by −0.002 for in-hospital AKI, +0.010 for 7-day AKI, and −0.003 for stages 2 and 3 AKI. There were 272 in-hospital deaths, including 87 without in-hospital AKI; 85 patients died within 7 days, including 33 without 7-day AKI. The three prespecified sensitivity analyses are reported in Supplemental Tables 11-13. Among 1,638 patients surviving at least 7 days, the discrimination ordering was preserved, and the corresponding AUROC estimates are reported in Supplemental Table 12. Performance patterns were similar during 2014 to 2019 (*n* = 1367) and 2020 to 2021 (*n* = 356), and no patient had COVID-19. Among the 406 patients with AKI, median onset was 4.11 days (IQR 1.89–8.91); 49 met criteria within 24 h, 128 within 48 h, and 281 within 7 days. Threshold-dependent operating characteristics are reported in Supplemental Tables 14-16.

## Discussion

In this external, head-to-head validation of 1723 emergency ICU patients, UCSD-Mayo showed the highest observed discrimination for all three AKI outcomes, but the absolute advantage was modest and, for 7-day AKI, not statistically significant versus AKI-RiSc (difference 0.029, 95%CI −0.006 to 0.064; *p* = 0.103). AKI occurred in 23.6% of the cohort, including 16.3% within 7 days and 9.8% with stages 2 and 3 disease, with a median onset of 4.11 days. Thus, the findings support the prespecified hypothesis that UCSD-Mayo would rank highest, yet do not establish categorical superiority.

These event rates are within the broad ranges reported in critically ill populations [3,30–38], although differences across studies may reflect case mix, creatinine sampling frequency, and availability of urine-output criteria. The relative performance of the scores may partly reflect alignment between their development populations and the present cohort. UCSD-Mayo was the only included instrument developed specifically in ICU patients [20], whereas AKI-RiSc, Bell, and APS were developed for emergency admissions, a one-year general-population horizon, and acute medical admissions, respectively [21–23]. Nevertheless, the AUROC of UCSD-Mayo in our cohort was lower than that reported in its original cohorts and in the COVID-19 validation by Fang et al. [39], underscoring the importance of reestablishing model performance when prediction tools are transported across settings and populations.

Calibration, reclassification, and decision-curve analyses provide a more nuanced interpretation of the observed ranking. After local recalibration, the four scores showed generally acceptable calibration in this cohort, but these results should not be interpreted as direct transport of the original published absolute risks because comparable original risk equations were not available for all scores and their prediction horizons differed. Reclassification generally favored UCSD-Mayo, although some improvements were small and the 7-day IDI versus AKI-RiSc remained uncertain. Decision-curve analyses likewise indicated that the relative net benefit of UCSD-Mayo varied across clinically relevant thresholds rather than remaining uniformly higher than that of the other scores. Taken together, these findings suggest a threshold-dependent and context-specific advantage rather than categorical superiority.

From a clinical perspective, the scores may be most useful as tools for structured risk awareness rather than treatment triggers. A higher predicted risk could prompt review of medication dosing and nephrotoxin necessity, closer creatinine monitoring, attention to hemodynamics and volume status, and consideration of nephrology consultation when otherwise indicated. Given that many AKI events occurred after admission rather than immediately at presentation, these tools may be more useful for informing surveillance intensity during the early hospital course than for triggering immediate therapy. Importantly, this retrospective validation provides no evidence that score-guided care improves patient outcomes.

Several limitations should be considered. First, the comparison was pragmatic rather than systematic and included only four transparent point scores that could be reconstructed from archived routine variables; ICU-specific models such as AKIpredictor [36] and biomarker-based tools were not evaluated, so UCSD-Mayo cannot be considered the optimal model among all available approaches. Second, the single-center retrospective design and exclusion of 1,020 of 2,743 screened patients (37.2%), whose individual-level records were no longer retrievable, leave the possibility of selection bias and limit generalizability to patients in whom all four scores were calculable. Third, the source data imposed several measurement limitations: admission eGFR may reflect acute kidney dysfunction rather than established chronic kidney disease; nephrotoxin exposure was captured as a whole-hospitalization indicator, introducing potential temporal leakage for an admission prediction model; AKI ascertainment was based on creatinine criteria without urine output; and the evaluated scores were developed for different prediction horizons. Finally, death may have competed with AKI ascertainment. There were 272 in-hospital deaths, including 87 patients without in-hospital AKI, and 85 deaths within 7 days, including 33 patients without 7-day AKI. Restriction to 7-day survivors preserved the discrimination ordering, but this sensitivity analysis does not substitute for a dedicated competing-risk time-to-event analysis.

In this Chinese emergency ICU cohort, UCSD-Mayo showed the highest observed discrimination for all three AKI outcomes, but its advantage was modest, was not statistically evident against AKI-RiSc for 7-day AKI, and was not uniform across performance metrics or decision thresholds. The four scores may support risk awareness and monitoring decisions, but none is ready to function as a stand-alone treatment rule. Prospective multicenter validation using harmonized prediction times, appropriate absolute-risk equations, and prespecified actionable thresholds, followed by clinical impact studies, is warranted.

## Supplementary Material

Revised_Supplemental_Materials_clean_260501445_final.docx

## Data Availability

The datasets used during the current study are available from the corresponding author on reasonable request.
