# Supplementary material for: External validation and comparative performance of four risk scores for acute kidney injury in critically ill adults
Source: Ren Fail. 2026 Oct 15;48(1):2728524. doi: 10.1080/0886022X.2026.2728524 (PMC13674667; doi:10.1080/0886022X.2026.2728524)
Supplement: Supplementary file 1 [file IRNF_A_2728524_SM1459.docx]

# **Supplementary Material List**

| **Supplemental Material** | **Description** |
| --- | --- |
| Supplemental Table 1 | UCSD-Mayo risk score |
| Supplemental Table 2 | AKI Risk Score (AKI-RiSc) |
| Supplemental Table 3 | Bell and Colleagues-AKI Risk Score |
| Supplemental Table 4 | Acute kidney injury prediction score (APS) |
| Supplemental Table 5 | Categorization of Primary Diagnoses by Disease in Patients With or Without AKI |
| Supplemental Table 6 | DeLong analyses comparing AUROC of the UCSD-Mayo risk score with that of the AKI-RiSc, Bell risk score and APS in predicting in-hospital AKI. |
| Supplemental Table 7 | DeLong analyses comparing AUROC of the UCSD-Mayo risk score with that of AKI-RiSc, Bell risk score and APS in predicting AKI within 7 days of admission. |
| Supplemental Table 8 | DeLong analyses comparing AUROC of the UCSD-Mayo risk score with that of AKI-RiSc, Bell risk score and APS in predicting more severe AKI (Stages 2 and 3). |
| Supplemental Table 9 | Cross-validated local calibration metrics for all scores and outcomes. |
| Supplemental Table 10 | IDI and categorical NRI comparing UCSD-Mayo with the other scores. |
| Supplemental Table 11 | Sensitivity analysis setting nephrotoxin exposure to zero. |
| Supplemental Table 12 | Sensitivity analysis restricted to patients surviving at least 7 days. |
| Supplemental Table 13 | Score discrimination by calendar period. |
| Supplemental Table 14 | Threshold-dependent performance for in-hospital AKI. |
| Supplemental Table 15 | Threshold-dependent performance for AKI within 7 days. |
| Supplemental Table 16 | Threshold-dependent performance for stage 2-3 AKI. |
| Supplemental Figure 1 | Participant flow |
| Supplemental Figure 2 | Apparent calibration plot for in-hospital AKI. |
| Supplemental Figure 3 | Apparent calibration plot for AKI within 7 days. |
| Supplemental Figure 4 | Apparent calibration plot for stage 2-3 AKI. |
| Supplemental Figure 5 | Decision curves for in-hospital AKI. |
| Supplemental Figure 6 | Decision curves for AKI within 7 days. |
| Supplemental Figure 7 | Decision curves for stage 2-3 AKI. |

**Supplemental Table 1. UCSD-Mayo risk score**

| **Category** | **Risk factor** | **Points** |
| --- | --- | --- |
| Chronic | Chronic kidney disease | 2 |
|  | Chronic liver disease | 2 |
|  | Congestive heart failure | 2 |
|  | Hypertension | 2 |
|  | Atherosclerotic coronary vascular disease | 2 |
| Acute | pH ≤ 7.30 | 3 |
|  | Nephrotoxin exposure | 3 |
|  | Severe infection/sepsis | 2 |
|  | Mechanical ventilation | 2 |
|  | Anemia | 1 |

Operational definitions in the present cohort: anemia was hemoglobin <9 g/dL (90 g/L) or hematocrit <27%. Nephrotoxins included amphotericin B, aminoglycosides, radiocontrast, chemotherapy, antiretrovirals, and NSAIDs other than aspirin; exposure was radiocontrast use, at least three concurrent nephrotoxins, or at least 3 days of aminoglycoside therapy. For the UCSD-Mayo and APS kidney-function items, the present cohort used admission eGFR calculated from the first admission creatinine; this was not a documented pre-existing CKD diagnosis.

**Supplemental Table 2. AKI Risk Score (AKI-RiSc)**

| **Predictor** | **Allocated Score** | | | | | |
| --- | --- | --- | --- | --- | --- | --- |
|  | 0 | 1 | 2 | 3 | 5 | 7 |
| Creatinine  (μmol/L) | < 100 |  | 100-149 |  | 150-249 | ≥ 250 |
| Bicarbonate  (mmol/L) | ≥ 20 | < 20 |  |  |  |  |
| Pulse  (beats/min) | < 100 |  | 100-119 | ≥ 120 |  |  |
| SBP (mmHg) | < 150 | ≥ 150 |  |  |  |  |
| DBP (mmHg) | < 90 | ≥ 90 |  |  |  |  |
| Age (years) | < 50 |  | ≥ 50 |  |  |  |

*SBP* systolic blood pressure, *DBP* diastolic blood pressure.

**Supplemental Table 3. Bell and Colleagues-AKI Risk Score**

|  | **Point** |
| --- | --- |
| Age(years) |  |
| 20-29 | 0 |
| 30-49 | 2 |
| 50-59 | 4 |
| 60-69 | 6 |
| 70-79 | 8 |
| >80 | 9 |
| eGFR(mL/min) |  |
| > 60 | 0 |
| 45-59 | 5 |
| 30-44 | 8 |
| < 30 | 16 |
| Diabetes mellitus | 3 |
| Heart failure | 4 |

**Supplemental Table 4. Acute kidney injury prediction score (APS)**

|  | **Score** | | | |
| --- | --- | --- | --- | --- |
|  | 0 | 1 | 2 | 3 |
| Age(years) | <60 |  | 60-79 | ≥80 |
| Respiratory rate  (breaths/min) | <20 | ≥20 |  |  |
| AVPU | alert |  |  | other |
| CKD stage 3a-5 | N | Y |  |  |
| Congestive heart  failure | N | Y |  |  |
| Diabetes mellitus | N |  | Y |  |
| Liver disease | N |  |  | Y |

AVPU: Alert, Verbal, Pain, Unresponsiveness score; CKD: chronic kidney disease.

**Supplemental Table 5. Categorization of Primary Diagnoses by Disease in Patients With or Without AKI**

| Disease Category | Whole cohort  (n=1723) | AKI  (n=406) | Non-AKI  (n=1317) |
| --- | --- | --- | --- |
| Cardiovascular diseases | 785 | 196(25%) | 589(75%) |
| Respiratory diseases | 611 | 139(23%) | 472(77%) |
| Gastrointestinal diseases | 80 | 12(15%) | 68(85%) |
| Neurological disorders | 70 | 18(26%) | 52(74%) |
| Infectious diseases | 48 | 13(27%) | 35(73%) |
| Renal diseases | 59 | 10(17%) | 49(83%) |
| Other | 40 | 12(30%) | 28(70%) |
| Metabolic/endocrine disorders | 22 | 3(14%) | 19(86%) |
| Trauma/surgical conditions | 8 | 3 (38%) | 5 (62%) |

AKI = acute kidney injury. Non-AKI = patients without AKI.

**Supplemental Table 6. DeLong analyses comparing AUROC of the UCSD-Mayo risk score with that of the AKI-RiSc, Bell risk score and APS in predicting in-hospital AKI.**

| **Comparators** | **Difference between AUROCs** | **95% CI** | **p-value** |
| --- | --- | --- | --- |
| UCSD-Mayo risk score − AKI-RiSc | 0.079 | 0.047–0.111 | <0.001 |
| UCSD-Mayo risk score − Bell risk score | 0.077 | 0.048–0.107 | <0.001 |
| UCSD-Mayo risk score − APS | 0.115 | 0.085–0.145 | <0.001 |

**Supplemental Table 7. DeLong analyses comparing AUROC of the UCSD-Mayo risk score with that of AKI-RiSc, Bell risk score and APS in predicting AKI within 7 days of admission.**

| **Comparators** | **Difference between AUROCs** | **95% CI** | **p-value** |
| --- | --- | --- | --- |
| UCSD-Mayo risk score − AKI-RiSc | 0.029 | −0.006–0.064 | 0.103 |
| UCSD-Mayo risk score − Bell risk score | 0.042 | 0.009–0.075 | 0.012 |
| UCSD-Mayo risk score − APS | 0.091 | 0.056–0.125 | <0.001 |

**Supplemental Table 8. DeLong analyses comparing AUROC of the UCSD-Mayo risk score with that of AKI-RiSc, Bell risk score and APS in predicting more severe AKI (Stages 2 and 3).**

| **Comparators** | **Difference between AUROCs** | **95% CI** | **p-value** |
| --- | --- | --- | --- |
| UCSD-Mayo risk score − AKI-RiSc | 0.059 | 0.016–0.103 | 0.008 |
| UCSD-Mayo risk score − Bell risk score | 0.072 | 0.031–0.113 | 0.001 |
| UCSD-Mayo risk score − APS | 0.144 | 0.104–0.184 | <0.001 |

**Supplemental Table 9. Cross-validated local calibration metrics.**

| **Outcome** | **Score** | **Slope (95% CI)** | **CITL (95% CI)** | **O/E (95% CI)** | **Brier score (95% CI)** |
| --- | --- | --- | --- | --- | --- |
| In-hospital AKI | UCSD-Mayo | 0.990 (0.829 - 1.158) | 0.000 (-0.117 - 0.121) | 1.000 (0.922 - 1.085) | 0.163 (0.153 - 0.174) |
| In-hospital AKI | AKI-RiSc | 0.988 (0.744 - 1.239) | -0.000 (-0.117 - 0.104) | 1.000 (0.917 - 1.078) | 0.173 (0.162 - 0.183) |
| In-hospital AKI | Bell | 0.982 (0.746 - 1.228) | -0.001 (-0.119 - 0.111) | 0.999 (0.916 - 1.084) | 0.172 (0.162 - 0.183) |
| In-hospital AKI | APS | 0.979 (0.675 - 1.292) | 0.000 (-0.109 - 0.116) | 1.000 (0.921 - 1.089) | 0.176 (0.167 - 0.188) |
| AKI within 7 days | UCSD-Mayo | 0.984 (0.798 - 1.201) | -0.001 (-0.138 - 0.125) | 1.000 (0.896 - 1.102) | 0.129 (0.118 - 0.139) |
| AKI within 7 days | AKI-RiSc | 0.978 (0.752 - 1.181) | -0.000 (-0.128 - 0.128) | 1.000 (0.902 - 1.106) | 0.130 (0.119 - 0.141) |
| AKI within 7 days | Bell | 0.981 (0.742 - 1.244) | -0.001 (-0.135 - 0.126) | 0.999 (0.896 - 1.105) | 0.131 (0.120 - 0.143) |
| AKI within 7 days | APS | 0.968 (0.626 - 1.329) | -0.001 (-0.133 - 0.120) | 0.999 (0.895 - 1.103) | 0.135 (0.123 - 0.146) |
| Stage 2-3 AKI | UCSD-Mayo | 0.994 (0.783 - 1.218) | -0.000 (-0.180 - 0.149) | 1.000 (0.856 - 1.133) | 0.084 (0.074 - 0.094) |
| Stage 2-3 AKI | AKI-RiSc | 0.979 (0.703 - 1.276) | -0.000 (-0.168 - 0.148) | 1.000 (0.862 - 1.137) | 0.086 (0.075 - 0.097) |
| Stage 2-3 AKI | Bell | 0.970 (0.690 - 1.274) | 0.000 (-0.171 - 0.153) | 1.000 (0.859 - 1.142) | 0.086 (0.075 - 0.097) |
| Stage 2-3 AKI | APS | 0.929 (0.275 - 1.616) | 0.000 (-0.165 - 0.142) | 1.000 (0.861 - 1.135) | 0.088 (0.077 - 0.099) |

*Predicted probabilities were obtained by stratified 10-fold cross-validated logistic mapping of each point score in this cohort. These values do not represent strict external calibration of the original absolute-risk equations.*

**Supplemental Table 10. Integrated discrimination improvement and categorical net reclassification improvement.**

| **Outcome** | **Comparison** | **Threshold** | **IDI (95% CI)** | **NRI (95% CI)** | **Event NRI** | **Nonevent NRI** |
| --- | --- | --- | --- | --- | --- | --- |
| In-hospital AKI | UCSD-Mayo vs AKI-RiSc | 23.6% | 0.055 (0.040 - 0.070) | 0.130 (0.070 - 0.192) | 0.138 | -0.008 |
| In-hospital AKI | UCSD-Mayo vs Bell | 23.6% | 0.054 (0.040 - 0.068) | 0.148 (0.090 - 0.207) | 0.165 | -0.017 |
| In-hospital AKI | UCSD-Mayo vs APS | 23.6% | 0.076 (0.063 - 0.089) | 0.213 (0.151 - 0.276) | 0.170 | 0.043 |
| AKI within 7 days | UCSD-Mayo vs AKI-RiSc | 16.3% | 0.011 (-0.003 - 0.025) | 0.087 (0.020 - 0.156) | 0.274 | -0.187 |
| AKI within 7 days | UCSD-Mayo vs Bell | 16.3% | 0.022 (0.010 - 0.033) | 0.091 (0.025 - 0.155) | 0.128 | -0.037 |
| AKI within 7 days | UCSD-Mayo vs APS | 16.3% | 0.044 (0.033 - 0.056) | 0.166 (0.095 - 0.237) | 0.146 | 0.020 |
| Stage 2-3 AKI | UCSD-Mayo vs AKI-RiSc | 9.8% | 0.024 (0.010 - 0.038) | 0.125 (0.043 - 0.207) | 0.314 | -0.189 |
| Stage 2-3 AKI | UCSD-Mayo vs Bell | 9.8% | 0.025 (0.013 - 0.037) | 0.113 (0.037 - 0.190) | 0.166 | -0.053 |
| Stage 2-3 AKI | UCSD-Mayo vs APS | 9.8% | 0.048 (0.036 - 0.060) | 0.202 (0.120 - 0.284) | 0.189 | 0.013 |

*Categorical NRI used the observed event rate for each outcome as the risk threshold.*

**Supplemental Table 11. Sensitivity analysis setting whole-stay nephrotoxin exposure to zero in UCSD-Mayo.**

| **Outcome** | **N** | **Events** | **Exposed** | **Original AUROC** | **Modified AUROC (95% CI)** | **Difference (95% CI)** |
| --- | --- | --- | --- | --- | --- | --- |
| In-hospital AKI | 1723 | 406 | 169 | 0.713 | 0.710 (0.682-0.738) | -0.002 (-0.012 - 0.007) |
| AKI within 7 days | 1723 | 281 | 169 | 0.690 | 0.700 (0.667-0.733) | 0.010 (0.000 - 0.019) |
| Stage 2-3 AKI | 1723 | 169 | 169 | 0.706 | 0.702 (0.661-0.744) | -0.003 (-0.018 - 0.010) |

**Supplemental Table 12. Seven-day AKI discrimination among patients surviving at least 7 days.**

| **Score** | **N** | **Events** | **AUROC** | **95% CI** |
| --- | --- | --- | --- | --- |
| UCSD-Mayo | 1638 | 229 | 0.677 | 0.640-0.714 |
| AKI-RiSc | 1638 | 229 | 0.656 | 0.617-0.695 |
| Bell | 1638 | 229 | 0.650 | 0.610-0.690 |
| APS | 1638 | 229 | 0.589 | 0.551-0.627 |

*There were 272 in-hospital deaths, including 87 without in-hospital AKI. Eighty-five patients died within 7 days: 33 without and 52 with 7-day AKI.*

**Supplemental Table 13. Discrimination by calendar period.**

| **Period** | **Outcome** | **Score** | **N** | **Events** | **AUROC** | **95% CI** |
| --- | --- | --- | --- | --- | --- | --- |
| 2014-2019 | In-hospital AKI | UCSD-Mayo | 1367 | 344 | 0.706 | 0.675-0.737 |
| 2014-2019 | In-hospital AKI | AKI-RiSc | 1367 | 344 | 0.633 | 0.599-0.667 |
| 2014-2019 | In-hospital AKI | Bell | 1367 | 344 | 0.634 | 0.600-0.669 |
| 2014-2019 | In-hospital AKI | APS | 1367 | 344 | 0.597 | 0.564-0.630 |
| 2014-2019 | AKI within 7 days | UCSD-Mayo | 1367 | 240 | 0.676 | 0.640-0.712 |
| 2014-2019 | AKI within 7 days | AKI-RiSc | 1367 | 240 | 0.659 | 0.621-0.696 |
| 2014-2019 | AKI within 7 days | Bell | 1367 | 240 | 0.644 | 0.605-0.683 |
| 2014-2019 | AKI within 7 days | APS | 1367 | 240 | 0.602 | 0.563-0.640 |
| 2014-2019 | Stage 2-3 AKI | UCSD-Mayo | 1367 | 144 | 0.704 | 0.659-0.748 |
| 2014-2019 | Stage 2-3 AKI | AKI-RiSc | 1367 | 144 | 0.653 | 0.606-0.699 |
| 2014-2019 | Stage 2-3 AKI | Bell | 1367 | 144 | 0.643 | 0.591-0.695 |
| 2014-2019 | Stage 2-3 AKI | APS | 1367 | 144 | 0.560 | 0.511-0.609 |
| 2020-2021 | In-hospital AKI | UCSD-Mayo | 356 | 62 | 0.750 | 0.686-0.815 |
| 2020-2021 | In-hospital AKI | AKI-RiSc | 356 | 62 | 0.647 | 0.566-0.728 |
| 2020-2021 | In-hospital AKI | Bell | 356 | 62 | 0.637 | 0.555-0.718 |
| 2020-2021 | In-hospital AKI | APS | 356 | 62 | 0.603 | 0.526-0.679 |
| 2020-2021 | AKI within 7 days | UCSD-Mayo | 356 | 41 | 0.765 | 0.691-0.838 |
| 2020-2021 | AKI within 7 days | AKI-RiSc | 356 | 41 | 0.687 | 0.587-0.787 |
| 2020-2021 | AKI within 7 days | Bell | 356 | 41 | 0.665 | 0.566-0.763 |
| 2020-2021 | AKI within 7 days | APS | 356 | 41 | 0.587 | 0.501-0.672 |
| 2020-2021 | Stage 2-3 AKI | UCSD-Mayo | 356 | 25 | 0.720 | 0.611-0.828 |
| 2020-2021 | Stage 2-3 AKI | AKI-RiSc | 356 | 25 | 0.623 | 0.495-0.752 |
| 2020-2021 | Stage 2-3 AKI | Bell | 356 | 25 | 0.589 | 0.469-0.708 |
| 2020-2021 | Stage 2-3 AKI | APS | 356 | 25 | 0.568 | 0.451-0.685 |

*No patient in either period had COVID-19.*

**Supplemental Table 14. Threshold-dependent performance for in-hospital AKI.**

| **Score** | **AUC** | **95% CI** | **p value** | **Optimal cutoff** | **Youden’s indices** | **Sensitivity** | **Specificity** | **PPV** | **NPV** | **FPR** | **FNR** | **Accuracy** |
| --- | --- | --- | --- | --- | --- | --- | --- | --- | --- | --- | --- | --- |
| UCSD-Mayo risk score | 0.713 | 0.685-0.740 | <0.001 | 8 | 0.339 | 70% | 64% | 38% | 87% | 36% | 30% | 66% |
| AKI-RiSc | 0.634 | 0.602-0.665 | <0.001 | 3 | 0.219 | 71% | 51% | 31% | 85% | 50% | 29% | 55% |
| Bell risk score | 0.635 | 0.603-0.667 | <0.001 | 13 | 0.217 | 69% | 53% | 31% | 85% | 47% | 31% | 57% |
| APS | 0.598 | 0.568-0.628 | <0.001 | 5 | 0.137 | 72% | 42% | 28% | 83% | 58% | 28% | 49% |

*Thresholds maximize Youden's index and are descriptive; they should not be interpreted as treatment thresholds.*

**Supplemental Table 15. Threshold-dependent performance for AKI within 7 days.**

| **Score** | **AUC** | **95% CI** | **p value** | **Optimal cutoff** | **Youden’s indices** | **Sensitivity** | **Specificity** | **PPV** | **NPV** | **FPR** | **FNR** | **Accuracy** |
| --- | --- | --- | --- | --- | --- | --- | --- | --- | --- | --- | --- | --- |
| UCSD-Mayo risk score | 0.690 | 0.658-0.723 | <0.001 | 8 | 0.301 | 69% | 61% | 26% | 91% | 39% | 31% | 62% |
| AKI-RiSc | 0.661 | 0.626-0.696 | <0.001 | 4 | 0.262 | 62% | 64% | 25% | 90% | 36% | 38% | 64% |
| Bell risk score | 0.648 | 0.612-0.684 | <0.001 | 14 | 0.241 | 65% | 59% | 24% | 90% | 41% | 35% | 60% |
| APS | 0.599 | 0.564-0.635 | <0.001 | 5 | 0.144 | 73% | 41% | 20% | 89% | 59% | 27% | 46% |

*Thresholds maximize Youden's index and are descriptive; they should not be interpreted as treatment thresholds.*

**Supplemental Table 16. Threshold-dependent performance for stage 2-3 AKI.**

| **Score** | **AUC** | **95% CI** | **p value** | **Optimal cutoff** | **Youden’s indices** | **Sensitivity** | **Specificity** | **PPV** | **NPV** | **FPR** | **FNR** | **Accuracy** |
| --- | --- | --- | --- | --- | --- | --- | --- | --- | --- | --- | --- | --- |
| UCSD-Mayo risk score | 0.706 | 0.665-0.746 | <0.001 | 9 | 0.315 | 57% | 75% | 20% | 94% | 25% | 43% | 73% |
| AKI-RiSc | 0.646 | 0.603-0.690 | <0.001 | 3 | 0.240 | 76% | 48% | 14% | 95% | 52% | 24% | 51% |
| Bell risk score | 0.634 | 0.587-0.681 | <0.001 | 21 | 0.250 | 43% | 82% | 21% | 93% | 18% | 57% | 78% |
| APS | 0.562 | 0.517-0.606 | <0.001 | 6 | 0.086 | 51% | 58% | 12% | 92% | 42% | 49% | 57% |

*Thresholds maximize Youden's index and are descriptive; they should not be interpreted as treatment thresholds.*

Supplemental Figure 1. Participant flow. Exclusion categories were recorded contemporaneously and may overlap; therefore, category counts are not additive.

Supplemental Figure 2. Apparent calibration for in-hospital AKI. Predicted probabilities were obtained from outcome-specific logistic models fitted in the full cohort, with each score as the sole predictor. Points and vertical bars show observed event rates in tenths of predicted probability with Wilson 95% confidence intervals; solid curves and shaded bands show LOESS smooths (span 0.657) with 95% confidence limits. Curves and confidence limits are shown only over the observed prediction range; gray histograms show the distribution of predicted probabilities.

Supplemental Figure 3. Apparent calibration for AKI within 7 days. Predicted probabilities were obtained from outcome-specific logistic models fitted in the full cohort, with each score as the sole predictor. Points and vertical bars show observed event rates in tenths of predicted probability with Wilson 95% confidence intervals; solid curves and shaded bands show LOESS smooths (span 0.657) with 95% confidence limits. Curves and confidence limits are shown only over the observed prediction range; gray histograms show the distribution of predicted probabilities.

Supplemental Figure 4. Apparent calibration for stage 2-3 AKI. Predicted probabilities were obtained from outcome-specific logistic models fitted in the full cohort, with each score as the sole predictor. Points and vertical bars show observed event rates in tenths of predicted probability with Wilson 95% confidence intervals; solid curves and shaded bands show LOESS smooths (span 0.657) with 95% confidence limits. Curves and confidence limits are shown only over the observed prediction range; gray histograms show the distribution of predicted probabilities.

Supplemental Figure 5. Decision-curve analysis for in-hospital AKI, including treat-all and treat-none strategies.

Supplemental Figure 6. Decision-curve analysis for AKI within 7 days, including treat-all and treat-none strategies.

Supplemental Figure 7. Decision-curve analysis for stage 2-3 AKI, including treat-all and treat-none strategies.
